# P-306. Associations between Intersectional Stigma and Long-Acting Injectable PrEP (LAI-PrEP) Willingness and Preference among Gay, Bisexual, and other Men who have Sex with Men (GBMSM)

**DOI:** 10.1093/ofid/ofaf695.526

**Published:** 2026-01-11

**Authors:** Jennifer L Glick, Danielle F Nestadt, Travis Sanchez, Iaah Lucas, Mariah Valentine-Graves, Tom Carpino, Duygu Islek, Kaitlyn Atkins, Sarah Murray, Stefan Baral, Supriya Sarkar, Leigh Ragone, Vani Vannappagari

**Affiliations:** Louisiana State University Health Sciences Center, Community Health Science & Policy, New Orleans, Louisiana; Johns Hopkins Bloomberg School of Public Health, Baltimore, Maryland; Emory University, Atlanta, GA; Emory University, Rollins School of Public Health, Atlanta, Georgia; Emory University, Rollins School of Public Health, Atlanta, Georgia; Duke Global Health Institute, Baltimore, Maryland; Emory University, Rollins School of Public Health, Atlanta, Georgia; Johns Hopkins University, Baltimore, Maryland; Johns Hopkins Bloomberg School of Public Health, Baltimore, Maryland; Johns Hopkins University, Baltimore, Maryland; ViiV Healthcare, Baltimore, Maryland; ViiV Healthcare, Baltimore, Maryland; ViiV Healthcare, Baltimore, Maryland

## Abstract

**Background:**

Gay, bisexual, and other men who have sex with men (GBMSM) in the United States (U.S.) are disproportionately impacted by the HIV epidemic. Long-acting injectable pre-exposure prophylaxis (LAI-PrEP) represents a novel HIV prevention strategy. However, stigma remains a known barrier to HIV-related prevention and care. We investigated the role of intersectional stigma (which manifests as discrimination) in LAI-PrEP preferences among U.S. cisgender GBMSM.

**Figure:**
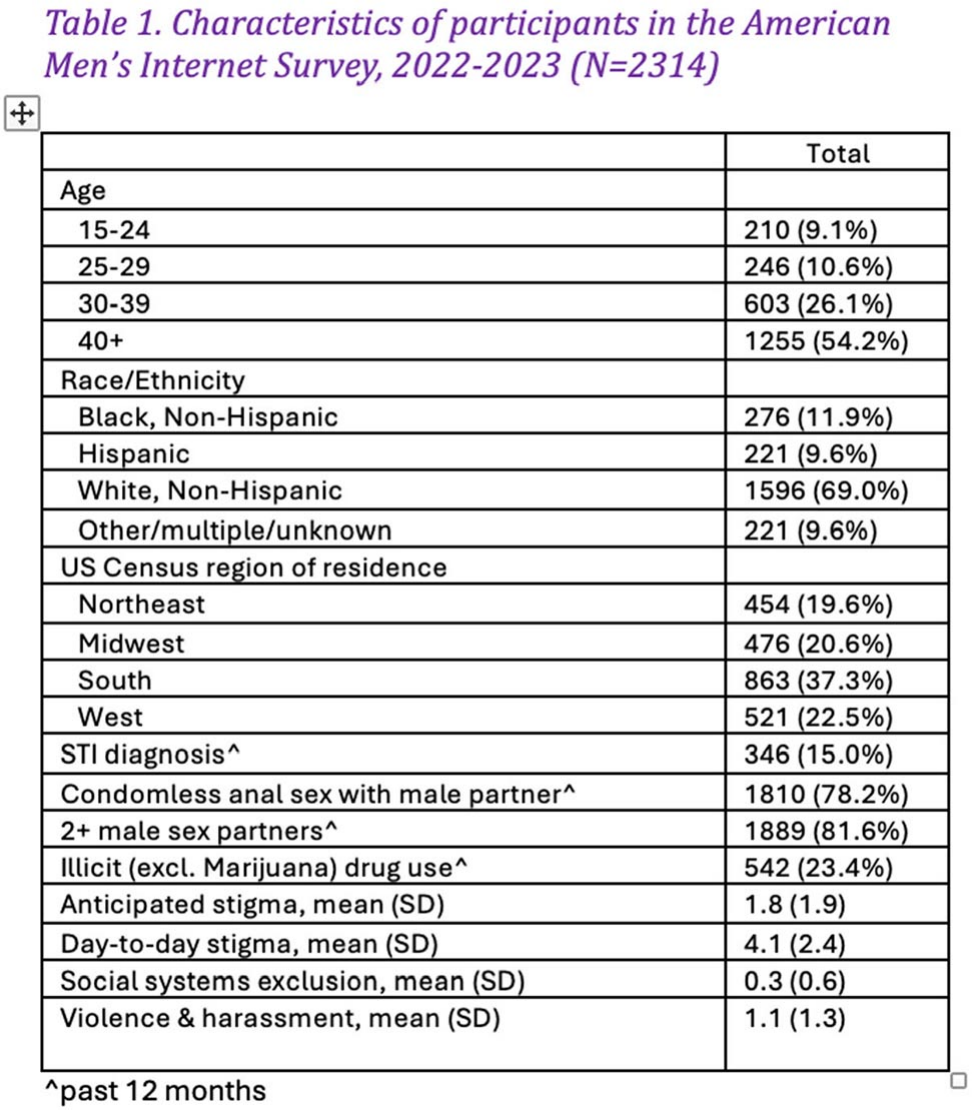

**Figure:**
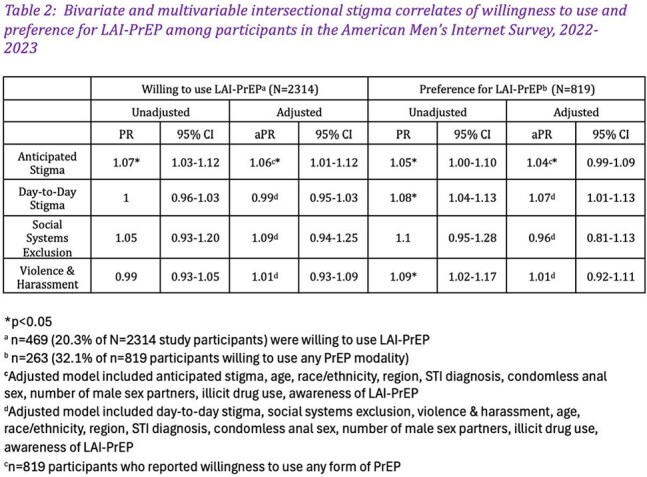

**Methods:**

The 2022 American Men’s Internet Survey (AMIS) enrolled cisgender GBMSM online between October 2022 and October 2023. Analyses were restricted to participants randomized (50/50) to receive intersectional stigma questions who reported no prior HIV diagnosis or past year PrEP use and provided a valid willingness to use LAI-PrEP response. PrEP modality preference analyses were further restricted to participants who reported willingness to use at least one PrEP modality. Using bivariate and multivariable adjusted Poisson regression models with robust variance, we used a modified 21-item version of the Intersectional Discrimination Index to examine associations between several forms of stigma—anticipated, day-to-day, social systems exclusion, and violence and harassment—and LAI-PrEP willingness and preference.

**Figure:**
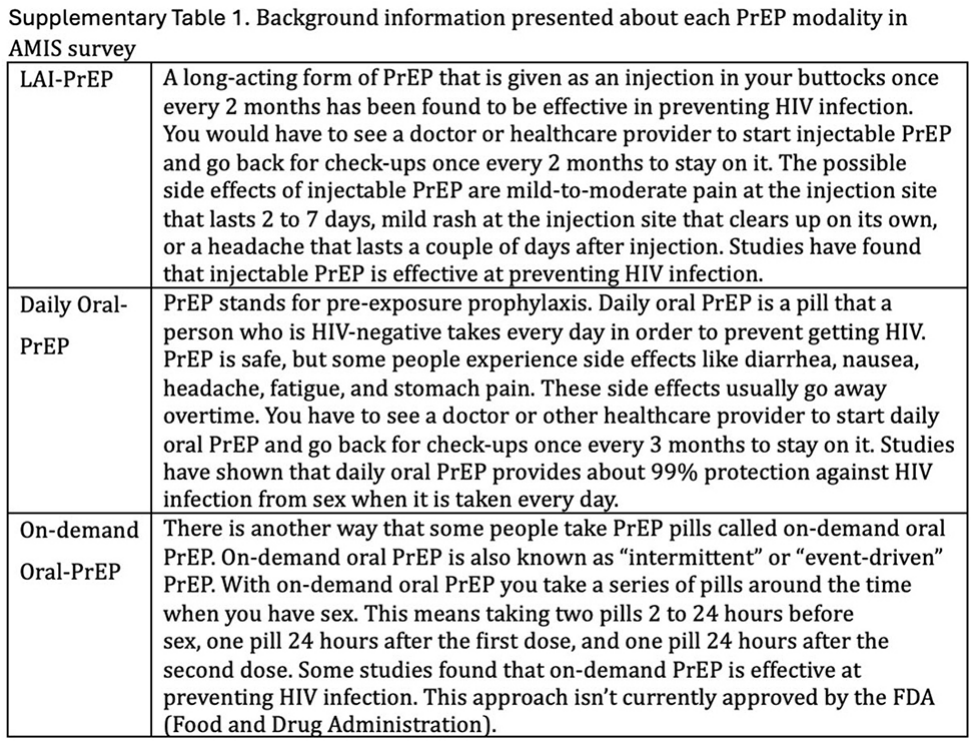

**Figure:**
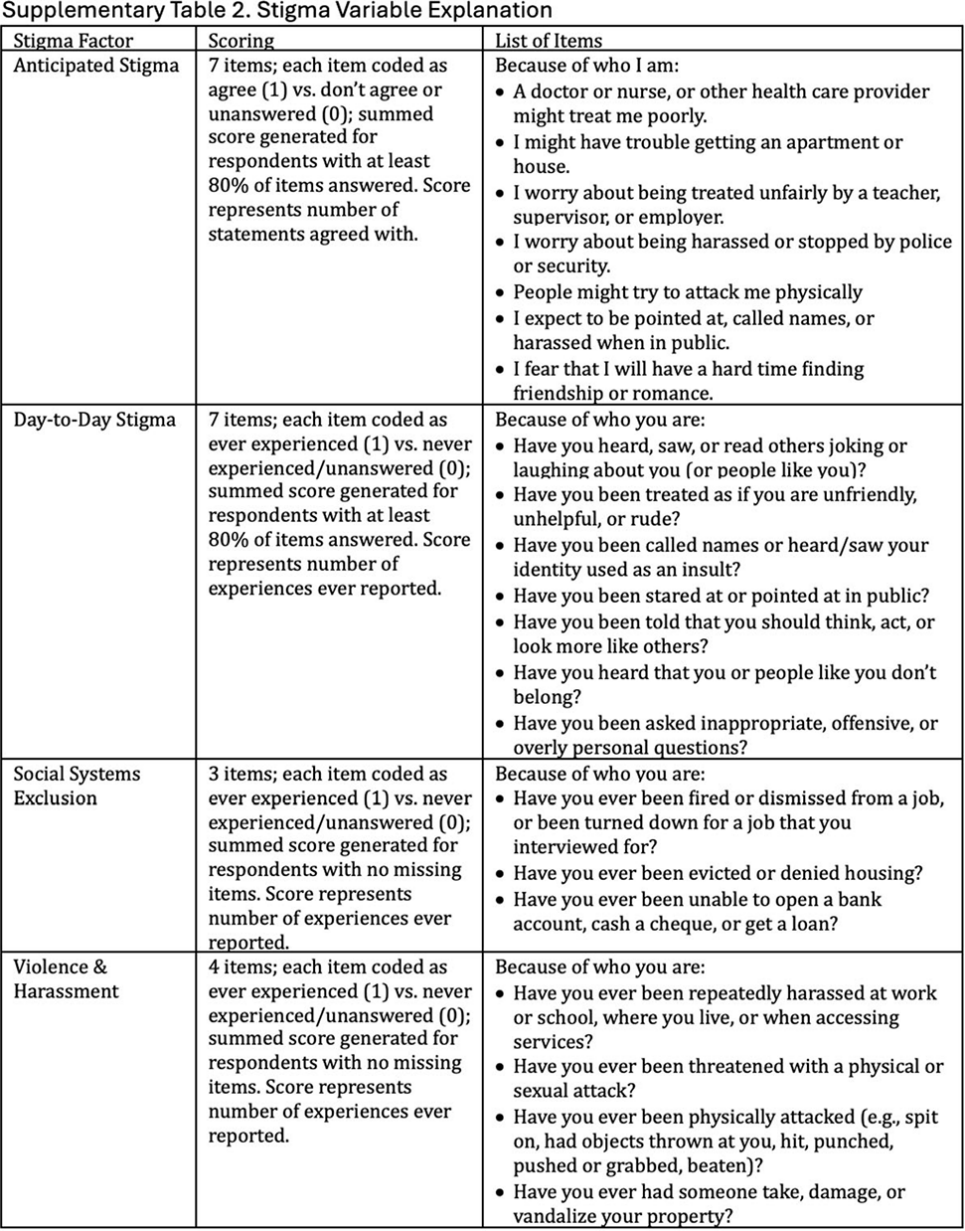

**Results:**

Among participants (N=2314), 819 (35.4%) were willing to use any form of PrEP and (469 [20.3%] were willing to use LAI-PrEP), among whom 263 (32.1%) reported a preference for LAI-PrEP over other PrEP modalities. On average, participants experienced 1.8 of the 7 anticipated discrimination items, 4.1 of the 7 day-to-day discrimination items, 0.3 of the 3 social systems exclusion items, and 1.1 of the 4 violence/harassment items (Table 1). Willingness to use LAI-PrEP was associated with anticipated stigma (aPR=1.06; 95% CI=1.01-1.12; p=0.01). Preference for LAI-PrEP vs. oral PrEP modalities was associated with day-to-day stigma (aPR=1.07; 95% CI=1.01-1.13; p=0.02) (Table 2).

**Conclusion:**

Given disproportionate HIV burden among GBMSM, HIV PrEP modality options (e.g., LAI-PrEP), which respond to diverse stigma concerns, combined with efforts to address intersectional stigma, are critical to ensure PrEP access, uptake, and adherence among GBMSM.

**Disclosures:**

Travis Sanchez, DVM, MPH, ViiV Healthcare, Inc.: Grant/Research Support Supriya Sarkar, PhD, MPH, ViiV Healthcare: Stocks/Bonds (Public Company) Leigh Ragone, MS, GSK: Stocks/Bonds (Private Company)|ViiV Healthcare: Employee Vani Vannappagari, MBBS, MPH, PhD, ViiV Healthcare: Full time Employee of ViiV Healthcare and owns GSK stock|ViiV Healthcare: Stocks/Bonds (Public Company)

